# Changes to the Autophagy-Related Muscle Proteome Following Short-Term Treatment with Ectoine in the Duchenne Muscular Dystrophy Mouse Model mdx

**DOI:** 10.3390/ijms26020439

**Published:** 2025-01-07

**Authors:** Eulàlia Gómez Armengol, Caroline Merckx, Hanne De Sutter, Jan L. De Bleecker, Boel De Paepe

**Affiliations:** Neuromuscular Reference Center and Department of Neurology, Ghent University Hospital, Corneel Heymanslaan 10, 9000 Ghent, Belgiumjan.debleecker@ugent.be (J.L.D.B.)

**Keywords:** muscular dystrophy, osmolytes, autophagy, mitophagy

## Abstract

The most severe form of muscular dystrophy (MD), known as Duchenne MD (DMD), remains an incurable disease, hence the ongoing efforts to develop supportive therapies. The dysregulation of autophagy, a degradative yet protective mechanism activated when tissues are under severe and prolonged stress, is critically involved in DMD. Treatments that harness autophagic capacities therefore represent a promising therapeutic approach. Osmolytes are protective organic molecules that regulate osmotic pressure and cellular homeostasis and may support tissue-repairing autophagy. We therefore explored the effects of the osmolyte ectoine in the standard mouse model of DMD, the mdx, focusing on the autophagy-related proteome. Mice were treated with ectoine in their drinking water (150 mg/kg) or through daily intraperitoneal injection (177 mg/kg) until they were 5.5 weeks old. Hind limb muscles were dissected, and samples were prepared for Western blotting for protein quantification and for immunofluorescence for an evaluation of tissue distribution. We report changes in the protein levels of autophagy-related 5 (ATG5), Ser366-phosphorylated sequestosome 1 (SQSTM1), heat shock protein 70 (HSP70), activated microtubule-associated protein 1A/1B-light chain 3 (LC3 II) and mammalian target of rapamycin (mTOR). Most importantly, ectoine significantly improved the balance between LC3 II and SQSTM1 levels in mdx gastrocnemius muscle, and LC3 II immunostaining was most pronounced in muscle fibers of the tibialis anterior from treated mdx. These findings lend support for the further investigation of ectoine as a potential therapeutic intervention for DMD.

## 1. Introduction

Duchenne muscular dystrophy (DMD) is a genetic muscle disorder that is characterized by progressive muscle wasting, which results in patients becoming wheelchair-bound during their teenage years [1,2]. As a consequence of its X-linked inheritance pattern, the condition predominantly affects males, with an estimated prevalence of 1 in 5000 live births [3]. DMD is caused by gene mutations in the *DMD* gene, which result in the loss of function of the scaffold protein dystrophin. This leads to the destabilization of the dystrophin-associated protein complex and, consequently, the accumulation of contraction-induced damage to muscle fiber membranes [4,5,6]. The increased permeability of muscle cells leads to an uncontrolled influx of calcium, which serves to further enhance the progressive degeneration of myofibers, mitochondrial rupture and the generation of reactive oxygen species [7,8]. In addition to chronic inflammation and accumulating oxidative stress, DMD muscle tissues suffer from dysfunctional autophagy, which contributes substantially to muscle weakness and wasting due to the inefficient removal of damaged or unwanted cellular components [9,10]. The key stages of macroautophagy are tightly regulated by a set of autophagy-related (ATG) proteins, which ensure the orchestration of process initiation, maturation and fusion with lysosomes [11,12]. The protein complex composed of ATG5-ATG12-ATG16L1 subunits represents a ubiquitin conjugation system that serves as an E3-like enzyme, which is imperative for the expansion and formation of autophagosomes. This ATG complex influences the lipidation of microtubule-associated protein 1A/1B-light chain 3 (LC3) and interacts with the multifunctional adaptor protein sequestosome 1 (SQSTM1), which are essential steps for autophagosome formation and fusion with lysosomes [10,11]. An important aspect of this process is mitophagy, i.e., the autophagic removal of damaged mitochondria. Mitophagy is mediated through the accumulation of PTEN-induced putative kinase 1 (PINK1) on the outer membrane of mitochondria that have lost their membrane potential. PINK1 subsequently recruits the E3 ubiquitin ligase parkin (PRKN) and SQSTM1, encapsulating damaged mitochondria within autophagosomes [13,14]. Mitophagy plays an essential role in the pathogenesis of DMD [9].

It is indisputable that the use of animal models has been pivotal in the study of DMD disease mechanisms and progression, as well as in the testing and evaluation of new therapeutic strategies. The most commonly utilized model is the dystrophin-deficient mouse, specifically the C57BL/10ScSn-Dmdmdx strain referred to as the mdx [15]. The mdx model carries a nonsense C-to-T transition in exon 23 of the *dmd* gene, which introduces a premature stop codon [15,16,17,18]. The absence of the dystrophin protein in this model results in the rapid onset of skeletal muscle degeneration and regeneration at the earliest stage. As the disease progresses, the rate of muscle wasting decelerates, resulting in a less severe disease phenotype to that observed in humans [8,15,16,18].

Despite advancements in molecular medicine, a cure for DMD remains elusive and the average life expectancy is reduced to the early forties [9,19]. Dystrophin gene replacement therapies and exon-skipping strategies have entered the clinic, and further molecular therapies are being pursued in clinical trials [20]. The current molecular strategies are, however, unable to cure patients. Their objective is to express a shorter, albeit partially functional dystrophin protein in patients, which improves the disease to phenotypical Becker muscular dystrophy. Additional limitations of these therapies are their mutation specificity, requiring a therapeutic design tailored to the individual patient, and the high costs [15,21]. Additionally, glucocorticoids need to be administered to patients with DMD to reduce inflammation-associated muscle damage. However, long-term treatment is associated with numerous adverse effects. Consequently, there is a need to investigate novel supportive treatments aimed at improving overall patient wellbeing and comfort.

It is well documented that osmotic stress contributes to the progressive deterioration of muscle tissues in DMD [22]. The absence of dystrophin from the muscle membrane renders muscle fibers more susceptible to hypo-osmotic shock, which results in hypercontraction and the leakage of soluble intracellular proteins from the muscle cells [23]. Moreover, the constitutive activation of the key osmotic regulator tonicity-responsive enhancer-binding protein (TonEBP), also known as nuclear factor of activated T-cells 5 (NFAT5), in DMD fibroblasts provides a potential explanation for the continuous buildup of fibrotic tissue [24]. The profound impact of osmotic stress in DMD suggests that protective osmolytes may serve as a promising supplementary treatment option. In this regard, taurine has been the subject of the most extensive research, and its beneficial effects have been observed in mouse models [25]. However, interference with murine development has also been identified, indicating a need for further investigation into alternative compounds.

Recently, we were the first to examine the effects of ectoine in the mdx [26]. Ectoine is a naturally occurring carboxamidine produced by extremophiles [27] and is compatible with cell metabolism even at high concentrations. Ectoine acts as a kosmotrope and protein stabilizer, boosting hydrophobic contacts and folding, and stabilizing cell membranes [28]. The established beneficial effects of ectoine include anti-inflammatory properties, which result from the deactivation of the pro-inflammatory transcription factor nuclear factor kappa-light-chain-enhancer of activated B cells (NFκB), and anti-oxidant properties, which arise from the elimination of hydroxyl radicals. Although functional improvement could not be observed, as measured by hanging grid and open field tests, our pioneering study noted an improved histopathology of skeletal muscle as well as anti-inflammatory effects in mdx after short-term ectoine treatment [26]. Histologic analyses of tibialis anterior (TA) muscle revealed significantly increased numbers of healthy muscle fibers and a decrease in the numbers of muscle fibers with central nuclei. Of the two fibrotic markers evaluated in TA muscle, transforming growth factor-β (TGF-β) levels were unaltered, while macrophage-derived osteopontin (SPP1) mRNA levels were significantly reduced in ectoine-treated mice. Taken together, these results point in the direction of subtle but potentially beneficial ectoine-mediated effects on muscle pathology. Given the potential of autophagy modulation as a therapeutic strategy for DMD and the beneficial effects attributed to osmolytes in this regard, we now present a follow-up study to evaluate the autophagic proteome of mdx subjected to the same ectoine treatment regimen as in the first study. Compensatory effects on the autophagic deficit in the mdx would enhance the utility of ectoine as a potential therapeutic agent for DMD.

## 2. Results

We first investigated the expression of autophagy-related proteins in skeletal muscle tissues from healthy controls (BL 10) and compared them to mdx mice. Secondly, we investigated the effect of short-term ectoine treatment on the expression/tissue distribution of autophagy-related proteins in mdx mice. As a treatment control group, we included mdx treated with intraperitoneal injections of saline. This study included muscles with predominantly oxidative and glycolytic characteristics, namely, the soleus (SOL) muscle and the extensor digitorum longus (EDL) muscle, respectively, and muscles with a mixed fiber type composition, including the gastrocnemius (GAS) and TA muscles. Quantitative differences were identified through Western blot techniques, while tissue distribution was studied through immunofluorescent microscopy.

### 2.1. Levels of Autophagy-Related Proteins of Skeletal Muscle of mdx Compared to Wild Type

**Figure 1:**
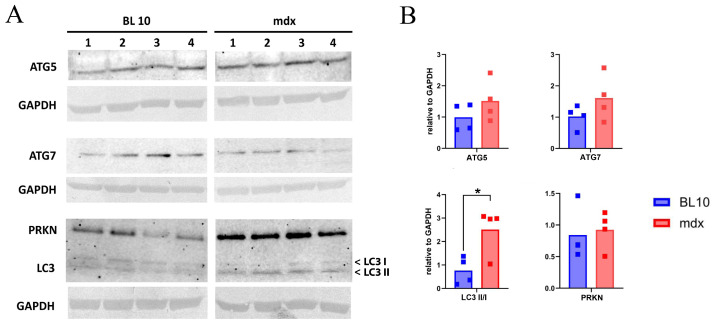
Protein levels in extensor digitorum longus muscle of 26-week-old mice. Total protein samples were prepared from four individual healthy control (BL 10 1–4) and four individual mdx (1–4) mice. A 20 µL sample (30 µg protein) was loaded in each lane. (**A**) Protein bands for autophagy-related 5 (ATG5), autophagy-related 7 (ATG7), microtubule-associated protein 1A/1B-light chain 3 (LC3), parkin (PRKN) and glyceraldehyde-3-phosphate dehydrogenase (GAPDH) as a loading control are shown. (**B**) Protein levels are reported as relative values to GAPDH. A significant difference can be observed in the ratio of phosphatidylethanolamine-conjugated LC3 (LC3 II) over unconjugated LC3 (LC3 I); *p* = 0.03 (*) between the BL 10 and mdx group, tested with an unpaired *t* test. Other protein levels are not significantly different. Graphs and statistical analyses were generated with GraphPad Prism 10.2.3. Full blots are provided as a supplement (Figure S1).

The levels of the proteins ATG5, ATG7, LC3 and PRKN were compared in the EDL from BL 10 and mdx mice at 26 weeks of age. The elevated ratios of LC3 II to LC3 I in mdx reached statistical significance (Figure 1).

### 2.2. Tissue Expression Patterns of Autophagy-Related Proteins

**Figure 2:**
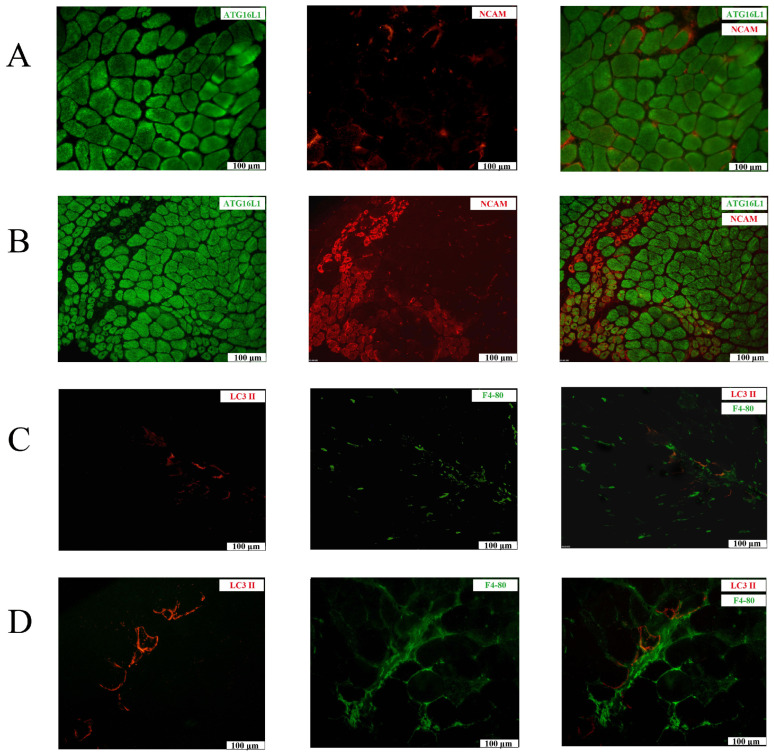
Immunofluorescence staining of mdx soleus muscle of 4-week-old mice (**A**) and tibialis anterior muscle of 12-week-old mice (**C**,**D**). (**A**) Staining for autophagy-related 16-like 1 (ATG16L1; AlexaFluor 488, green) and neural cell adhesion molecule (NCAM; CY3, red) in a BL 10 control (**A**) and an mdx mouse (**B**) shows strong sarcoplasmic staining in the majority of muscle fibers. Strong NCAM staining is present in a cluster of small perifascicular muscle fibers in the mdx section, indicating that these are regenerating muscle fibers, and from these muscle fibers, ATG16L1 staining is absent. Staining for microtubule-associated protein 1A/1B-light chain 3 conjugated to phosphatidylethanolamine (LC3 II; CY3, red) and macrophages (F4-80; AlexaFluor 488, green) are shown in a BL 10 control (**C**) and an mdx mouse (**D**). (**C**) In a healthy control, LC3 II staining is mostly absent, and a few scattered macrophages can be observed. (**D**) In an mdx section, sarcolemmal LC3 II staining localizes to muscle fibers in perifascicular regions. Macrophages are abundant in this microscopic field, indicated by bright green fluorescence. The diffuse and weaker staining that can be observed in the connective tissue accumulating in the perifascicular region can be considered nonspecific. Scale bars = 100 µM.

In SOL and TA muscles from BL 10, homogeneous sarcoplasmic staining for the ATG16L1 subunit of the E3-like enzyme complex ATG12–ATG5-ATG16L1 could be observed (Figure 2A). Sections were co-stained for neural cell adhesion molecule (NCAM), a marker for regenerating muscle fibers. Reduced expression levels of ATG16L1 were present in regenerating muscle fibers, with the greatest reduction observed in clusters of small fibers (Figure 2B). In mdx tissues, immunostaining for LC3 II was increased compared to in BL 10 (Figure 2C,D). In mdx, LC3 II staining was most often discontinuous on the sarcolemma of subsets of muscle fibers. The grouping of small LC3 II-positive fibers in perifascicular regions could at times be observed (Figure 2D). Strong sarcoplasmic staining for heat shock protein 70 (HSP70) chaperones was observed in necrotic muscle fibers (Supplementary Figure S2A), and a subset of muscle fibers stained strongly for SQSTM1, with the majority being NCAM-negative (Supplementary Figure S2B).

### 2.3. The Impact of Ectoine Treatment on the Levels of Autophagy-Related Proteins

**Figure 3:**
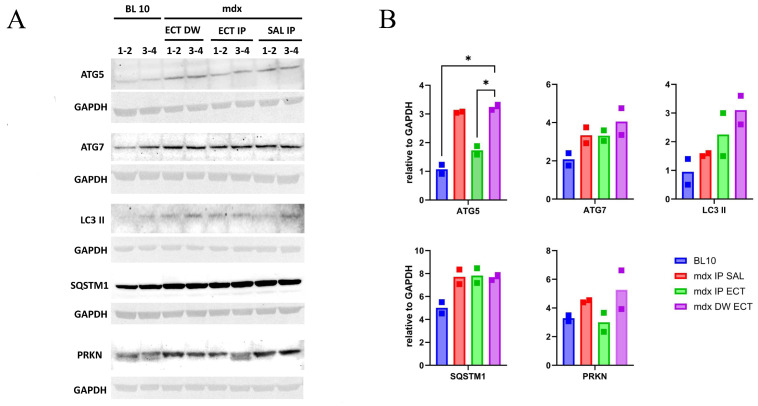
Protein levels in extensor digitorum longus muscle of untreated and ectoine-treated mice. Two total protein samples were prepared per group by pooling muscles from two individual mice. A 20 µL sample (30 µg protein) was loaded in each lane. (**A**) Protein bands for autophagy-related 5 (ATG5), autophagy-related 7 (ATG7), microtubule-associated protein 1A/1B-light chain 3 conjugated to phosphatidylethanolamine (LC3 II), sequestosome 1 (SQSTM1), parkin (PRKN) and glyceraldehyde-3-phosphate dehydrogenase (GAPDH) as a loading control are shown in control black 10 mice (BL 10 1–4), and mdx treated with ectoine in their drinking water (ECT DW 1–4), ectoine administered via intraperitoneal injection (ECT IP 1–4), and intraperitoneal saline (SAL IP 1–4), serving as a treatment control. (**B**) Protein levels are reported as values relative to glyceraldehyde-3-phosphate dehydrogenase (GAPDH). Significant changes can be observed for ATG5 levels, with an adjusted *p* = 0.02 between BL 10 and mdx DW ECT (*), and *p* = 0.04 between mdx IP ECT and mdx DW ECT (*), tested with the Brown–Forsythe test and Welch ANOVA for multiple comparisons. Graphs and statistical analyses were generated with GraphPad Prism 10.2.3. Full blots are provided as a supplement (Figure S1).

The levels of the proteins ATG5, ATG7, LC3 II, SQSTM1 and PRKN were evaluated in EDL muscle from the BL 10 and mdx treatment groups (Figure 3). The levels of ATG5 were significantly higher in the mdx group treated with ectoine in their drinking water (DW ECT) compared to the BL 10 group. The levels of ATG5 were significantly lower in the mdx group treated with ectoine via intraperitoneal injection (IP ECT) compared to the DW ECT group. The levels of other proteins did not significantly differ between groups. In regard to PRKN, a double band was observed in both BL 10 samples and in one of the ECT IP samples, which may be indicative of post-translational modifications. Only the upper band, that was present in all samples, was included for quantification.

**Figure 4:**
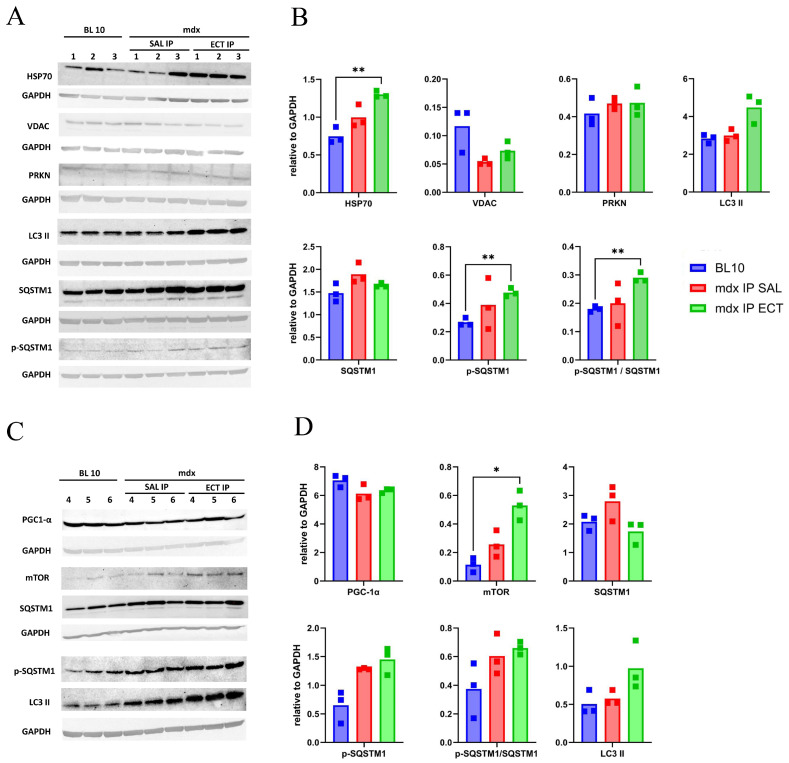
Protein levels in gastrocnemius muscle of untreated and ectoine-treated mice. Total protein samples were prepared from two sets of three individual mice per group: control black 10 mice (BL 10 1–3, 4–6), mdx administered with saline intraperitoneally (SAL IP 1–3, 4–6) and mdx administered with ectoine intraperitoneally (SAL IP 1–3, 4–6). A 20 µL sample (30 µg protein) was loaded in each lane. (**A**,**C**) Protein bands for heat shock protein 70 (HSP70), voltage-dependent anion channels (VDACs), parkin (PRKN), microtubule-associated protein 1A/1B-light chain 3 conjugated to phosphatidylethanolamine (LC3 II), sequestosome 1 (SQSTM1), SQSTM1 phosphorylated on its Ser366 residue (p-SQSTM1), peroxisome proliferator-activated receptor-gamma coactivator 1α (PGC-1α), mammalian target of rapamycin (mTOR) and glyceraldehyde-3-phosphate dehydrogenase (GAPDH) as a loading control are shown (**B**–**D**). Protein levels are reported as relative values to GAPDH. In the first sample set, significant changes can be observed between BL 10 and mdx IP ECT in HSP70 (adjusted *p* = 0.009 **) and p-SQSTM1 levels (adjusted *p* = 0.003); the latter remains significantly different when calculated as a ratio of phosphorylated over total SQSTM1 protein levels (adjusted *p* = 0.006 **). In the second sample set, mammalian target of rapamycin (mTOR) levels are significantly higher in IP ECT compared to BL 10 (adjusted *p* = 0.02 *). A significant increase in pSQSTM1 cannot be confirmed; however, the levels of the proteins p-SQSTM1 and LC3 II do display a tendency to increase. Graphs and statistical analyses with the Brown–Forsythe test and Welch ANOVA for multiple comparisons were generated with GraphPad Prism 10.2.3. Full blots are provided as a supplement (Figure S1).

The levels of HSP70, voltage-dependent anion channels (VDACs), PRKN, LC3 II, SQSTM1, Ser366-phosphorylated SQSTM1 (p-SQSTM1), peroxisome proliferator-activated receptor-gamma coactivator 1α (PGC-1α) and mammalian target of rapamycin (mTOR) were evaluated in GAS muscle from BL 10 and mdx treated with saline or ectoine through intraperitoneal injection (Figure 4). In a first set of three mice per group (Figure 4A,B), the levels of HSP70 chaperones were significantly elevated in mdx IP ECT in comparison to BL 10. Furthermore, p-SQSTM1 levels were significantly elevated between these groups, and this increase remained significant when calculated as a ratio of phosphorylated to total SQSTM1 protein levels. The levels of VDAC were not significantly diminished in the mdx group, indicating that mitochondrial content was preserved, and levels remained unaltered in the treated mice. A significant induction of p-SQSTM1 could not be confirmed in a second set of three mice per group (Figure 4C,D); however, in this set, the levels of p-SQSTM1 and LC3 II also tended to increase with ectoine treatment.

**Figure 5:**
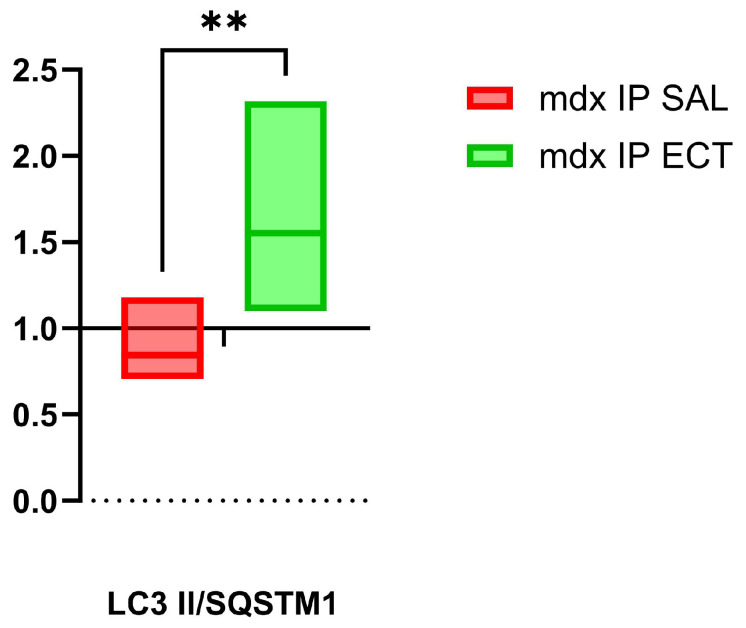
Autophagic efficiency responds to ectoine treatment in mdx. Theoretical autophagic efficiency is calculated as the ratio of microtubule-associated protein 1A/1B-light chain 3 conjugated to phosphatidylethanolamine (LC3 II) over sequestosome 1 (SQSTM1) as relative to the ratio observed in healthy control mice (n = 6) set to 1.0, presented as boxplots showing the median and interquartile range. In comparison to control mdx treated with intraperitoneal saline (IP SAL n = 6), autophagic efficiency is significantly higher in mdx treated with ectoine (IP ECT n = 6), as demonstrated by performing an unpaired *t*-test (*p* = 0.003 **). Graphs and statistical analyses were generated with GraphPad Prism 10.2.3.

As a measure of autophagic efficiency, we calculated the ratio of LC3 II to SQSTM1 in individual mice of each group. For the GAS muscle, the sample size could be increased to six mice per group by combining the Western blotting results from the two sample sets, setting the mean value for BL 10 to 1.0 for each sample set. The ratios of LC3 II/SQSTM1 were 0.85 ± 0.18 in mdx IP SAL (n = 6) and increased significantly to 1.55 ± 0.42 in mdx IP ECT (n = 6), as determined by an unpaired *t*-test (*p* = 0.003) (Figure 5). A tendency for increased ratios was also noticeable in the EDL muscle, with a value of 0.63 ± 0.60 in mdx IP SAL (n = 2), increasing to 1.75 ± 0.57 in mdx IP ECT (n = 2) and 1.20 ± 0.07 in mdx DW ECT (n = 2).

### 2.4. The Impact of Ectoine on the Tissue Expression Patterns of Autophagy-Related Proteins

**Figure 6:**
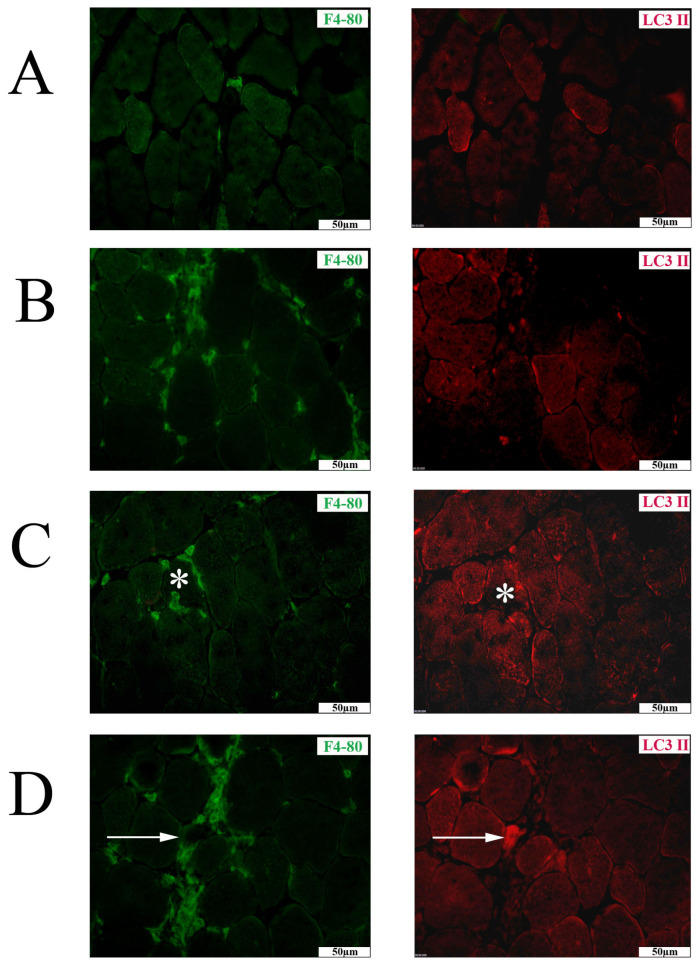
Immunofluorescence co-staining with F4/80 antibody, which stains macrophages (AlexaFluor 488, green), and 1A/1B-light chain 3 conjugated to phosphatidylethanolamine (LC3 II, CY3, red) in tibialis anterior muscle. In a healthy control (**A**) and mdx treated with intraperitoneal saline (**B**), infrequent partial sarcolemmal LC3 II staining is observed on certain muscle fibers. (**C**) In an mdx treated with intraperitoneal ectoine, macrophage staining localizes a necrotic invaded muscle fiber, which is LC3 II-negative (asterisk). (**D**) In an mdx treated with ectoine in its drinking water, macrophages are abundant, and a small muscle fiber surrounded by an inflammatory infiltrate is strongly LC 3 II-positive (arrow). Scale bars = 50 µm.

As anticipated, muscle-infiltrating inflammatory cells were more abundant in the TA from mdx compared to BL 10. However, notable variations in macrophage abundance were observed between individual mdx mice. Nevertheless, a tendency for larger numbers of macrophages was observed in the TA from mdx ECT DW (Figure 6D). The TA from age-matched BL 10 was predominantly negative for LC3 II, with the exception of the partial staining of the sarcolemma of subsets of muscle fibers (Figure 6A). In mdx tissues, there was an increase in staining for LC3 II on muscle fibers. The majority of necrotic invaded muscle fibers exhibited negative staining for LC3 II (Figure 6C), while subsets of small muscle fibers demonstrated strong LC3 II staining. These fibers were more frequently observed in mdx ECT DW (Figure 6D). The staining patterns of HSP70 and SQSTM1 were similar between untreated mdx and the different treatment groups, and thus, no further analysis was conducted on this data set.

### 2.5. Impact of Ectoine Treatment on Autophagy-Related Protein Levels in Mitochondrial Fractions

**Figure 7:**
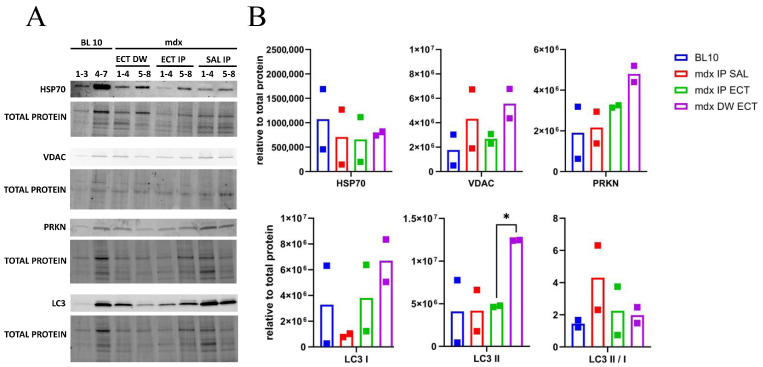
Mitochondrial protein levels in soleus muscle of untreated and ectoine-treated mice. Two mitochondria-enriched protein samples were prepared per group by pooling soleus muscle from four individual mice (except for the first BL 10 sample, which was prepared from three mice). (**A**) Protein bands for heat shock protein 70 (HSP70), voltage-dependent anion channels (VDACs), parkin (PRKN), microtubule-associated protein 1A/1B-light chain 3 (LC3) and the corresponding total protein stain are shown in control black 10 mice (BL 10 1–7 and in mdx) intraperitoneally administered with saline (SAL IP 1–8) or ectoine (ECT IP 1–8), or ectoine in the drinking water (ECT DW 1–8). (**B**) Protein levels are reported as relative values normalized to total protein content. Significant changes can be observed for LC3 II levels, with an adjusted *p* = 0.01 between mdx IP ECT and mdx DW ECT (*); significance does not remain when calculated as a ratio of LC3 II over I. Graphs and statistical analyses with the Brown–Forsythe test and Welch ANOVA for multiple comparisons were generated with GraphPad Prism 10.2.3. Full blots are provided as a supplement (Figure S1).

To assess mitophagy in ectoine-treated mdx, mitochondrial protein fractions were prepared from soleus (SOL) muscle and the levels of HSP70, VDAC, PRKN and LC3 were quantified (Figure 7). For normalization purposes, total protein levels determined through stain-free blot technology were used after the methodology was tested for robustness in comparison to housekeeping protein levels. Adjusted volumes for GAPDH were compared using an unpaired two-sample *t*-test and were found to be unaltered in mdx compared to BL 10 (Supplementary Figure S3). Significantly elevated LC3 II levels were observed between mdx IP ECT and mdx DW ECT, yet the difference was no longer significant when calculated as a ratio of LC3 II over LC3 I. Attempts to quantify SQSTM1 in mitochondrial extracts were unsuccessful, precluding a comparison between the treatment groups.

## 3. Discussion

In addition to enhancing the levels of functional dystrophin protein, targeting other DMD-associated pathogenic mechanisms represents a valuable therapeutic strategy in its own right. In this regard, immunosuppressive therapy has been demonstrated to be unsurpassable for DMD disease management. It is necessary to restrain the inflammatory processes that aggravate muscle tissue damage, while preserving the beneficial reparative and myogenic functions that accompany these processes. An additional treatment strategy for DMD would be to address the catabolic processes of proteins and tissue recovery deficits in a balanced and targeted manner. In the event of severe and persistent stress on muscle tissue, the autophagic removal of damaged cell material is vital for recovery. However, dystrophin deficiency has been linked to the insufficient activation of restorative programs [9]. In this context, the advantageous effects of osmolytes extending beyond osmoregulation are of interest. The administration of taurine, the most extensively studied osmolyte in the mdx mouse, has been demonstrated to enhance autophagic capabilities in muscle tissues [29]. This observation prompted us to investigate the expression of autophagy-related proteins in mdx mice treated with ectoine. The present explorative study reports altered expression levels in young mdx mice that were treated with ectoine during the most active phase of skeletal muscle degeneration and regeneration. While these alterations were limited, they could nonetheless indicate a favorable stimulation of autophagy.

For autophagy to be effective, the activities of a multitude of involved factors must align with the cell’s requirements. ATG proteins play a pivotal role in these processes. The significantly elevated ATG5 protein levels we observed in the EDL of mdx treated with ectoine in their drinking water could be considered a protective mechanism. A decline in ATG5 has been linked to age-related cellular damage [30]; conversely, the overexpression of ATG5 has been demonstrated to extend lifespan in mice [31]. Moreover, in vitro studies utilizing ATG5 siRNA demonstrated autophagy dysregulation and interference with myogenic differentiation [32]. Skeletal muscle is a dynamic tissue, and it seems that the expression of ATG proteins requires regulation during muscle tissue remodeling. We report the loss of ATG16L1 expression in small regenerating muscle fibers, which further implicates ATG protein complex reorganization in myogenesis. In mice, an *Atg16L1* gene defect has been demonstrated to cause muscle atrophy [33]. Additionally, ATG16L1 has been shown to play a role in maintaining cellular homeostasis through its involvement in regulating inflammatory responses [34]. The intricate regulation of ATG proteins in muscle remodeling is further achieved through the precise modulation of gene expression by non-coding microRNAs (miRNAs). The elevated levels of miR-106b observed in DMD [35] are of particular interest, due to deleterious effects on muscle tissue regeneration [36]. Consequently, it has been proposed as a potential therapeutic target [37]. MiR-106b interacts with numerous autophagy-related genes, thereby playing an indispensable role in autophagy regulation. Direct interaction with the ATG16L1 mRNA 2′UTR has been demonstrated [38].

The dysregulation of autophagic markers in mdx has been described as a complex phenomenon that is age- and tissue-dependent [9]. In this regard, the relatively young age of the mice in this study is of particular importance, as this represents the most active disease phase in terms of skeletal muscle degeneration and regeneration. In accordance with the aforementioned characteristics of young mice, our earlier study revealed a significant 2.2-fold increase in HSP70 chaperone levels in the TA from mdx compared to BL 10 specifically at the age of 4 weeks, which later returned to the levels of healthy controls [39]. HSP70 chaperones are crucial decision-making proteins that determine whether proteins are directed toward repair or destruction [40]. They work in close collaboration with autophagic factors. In the present study, we observed a significant increase in HSP70 protein levels in mdx treated with intraperitoneal ectoine, which could potentially be beneficial for tissue recovery. In addition, HSP70 exerts a protective effect on mitochondria. Tumoral cells have been described to possess elevated mitochondrial HSP70 levels, and in these cells, HSP70 inhibition disturbs mitochondrial function through the accumulation of polyubiquitylated proteins [41]. In contrast to the elevated total HSP70 levels observed in the TA, we did not observe a concomitant increase in mitochondrial HSP70 levels in the SOL from ectoine-treated mdx. This suggests that the mitochondrial chaperone machinery may not be activated.

A crucial autophagy substrate selector is LC3, the altered activation of which has been documented in mdx (Table 1) [42,43,44,45,46,47,48,49,50]. The data show differences with age and between muscles tested. We observed significantly increased LC3II/I ratios in the EDL muscle of 26-week-old mdx when compared to age-matched healthy controls. It is noteworthy that mitochondrial LC3 II levels were significantly elevated in the SOL muscle from mdx treated with ectoine in their drinking water, which could indicate the activation of mitophagy. Concomitant higher levels of LC3 I did, however, result in an unaltered LC3 II/I ratio.

Altered levels of the autophagy receptor SQSTM1 have also been demonstrated in mdx muscle tissues (Table 1). Nevertheless, the interpretation of these findings is challenging given that SQSTM1 regulates its own degradation. Consequently, elevated SQSTM1 levels may be indicative of dysfunctional autophagy, resulting from its accumulation and impaired clearance. Furthermore, the autophagic deficit in DMD patients and mdx may not be reflected by concomitant decreases in gene and protein expression levels. Similar or significantly decreased SQSTM1 mRNA levels were consistently accompanied by significantly increased protein levels in mdx quadriceps [14], TAs [46] and diaphragms [51]. In our study reporting on 5.5-week-old mdx, mdx SQSTM1 levels did not differ from BL 10 controls; however, the increase in Ser366-phosphorylated SQSTM1 levels in ectoine-treated mdx reached significance in one of two GAS muscle sample sets tested. This is a noteworthy observation as post-translational regulatory mechanisms may also influence autophagic processes. Ser366 is a primary phosphorylation site for the TANK-binding kinase 1 (TBK1), a kinase situated at the nexus of immunity and autophagy. TBK1 plays a pivotal role in mitophagy, whereby it is recruited to damaged mitochondria [52] and activates their selective removal through its association with autophagy adaptors including SQSTM1 [53]. The phosphorylation of Ser366 renders SQSTM1 more stable [54], which may be beneficial to the muscle tissue in the early stages of active disease. It is also noteworthy that *TBK1* gene defects have been associated with neurodegenerative disorders [55]. It is not so much increased levels of LC3 II and SQSTM1 proteins but rather their coordinated activities that appear crucial in the control of muscle tissue repair and differentiation. In vitro experiments have determined that autophagic activities in myoblasts are low and accelerate upon differentiation to myotubes, mostly in the earliest phase, with increased LC3 II and decreased SQSTM1 levels. Later in the process, LC3 II decreases and SQSTM1 increases [56]. An appropriate response to restore the chronic muscle tissue damage associated with dystrophin loss would require optimal autophagic functionality characterized by elevated levels of LC3 II in conjunction with diminished levels of SQSTM1. We report significantly higher LC3 II to SQSTM1 ratios in the GAS muscle from ectoine-treated mdx, supporting the hypothesis that ectoine supplementation may alleviate progressive muscle damage in mdx mice by compensating for the autophagic deficit, potentially improving the clearance of defective cellular components. Mechanistic research is, however, needed to confirm or refute this hypothesis, as the descriptive data we present in this study are only indicative of increased autophagic efficiency. International guidelines have been issued stressing the need to assess the rate at which autophagy substrates are delivered to lysosomes and degraded, termed the autophagic flux, in detail. Monitoring LC3 II levels at single timepoints and/or without examining turnover is not sufficient and needs to be compared to autophagy deficiency induced by autophagy inhibitors [57].

The small size of limb muscles at this early developmental stage constrained the number of autophagic factors that could be quantified. For all muscles except the GAS, it was necessary to combine material from multiple individuals to obtain sufficient protein for repeated Western blots. We also need to acknowledge that we were unable to include untreated mdx as a control group and only provide quantitative data on saline-injected mdx controls. Another limitation of this study is that important regulatory mechanisms associated with autophagy regulation were not fully investigated. There is a need to further evaluate the effects of ectoine on key regulatory pathways. The increase we observed in mTOR levels in mdx IP ECT is of interest, as mTOR is a potent modulator of autophagy via its downstream targets phosphatidylinositol 3 kinase (PI3K) and kinase AKT (AKT) [58]. In the mdx, the stimulation of AKT signaling has been observed to be beneficial, partly restoring membrane integrity by inducing utrophin and integrin expression [59]. Furthermore, the PI3K/AKT/mTOR pathways can activate PGC-1α, a protector of mitochondrial activity within the cell through the stimulation of mitochondrial biogenesis and restorative mitophagy [60]. Mdx overexpressing PGC-1α display an improved phenotype [61]. In this study, we could not observe changes in PGC-1α levels, nor did we note mitochondrial proliferation after ectoine treatment. Informed by the results of this explorative study, future studies can be designed to build a more comprehensive understanding of the mechanisms underlying ectoine-induced effects, which could lead to better-targeted and more effective therapeutic regimens to be tested in the mdx. Considering the important differences between the animal model and human disease, the effects of ectoine could be very different in patients and would need to be investigated. Our observation that ectoine treatment improves the balance between the autophagic effectors LC3 II and SQSTM1 in mdx GAS muscle lend support for the further investigation of this osmolyte as a potential therapeutic intervention for DMD.

## 4. Materials and Methods

### 4.1. Animal Care, Treatment and Muscle Sampling

C57BL/10ScSn-Dmdmdx/J (mdx) mice and C57BL/10SnJ control mice (BL 10) were housed at the central animal facility of Ghent University. All experimental procedures were conducted in accordance with the ARRIVE guidelines and were approved by the Animal Ethics Committee of Ghent University. All animals had access to food and water ad libitum.

Mdx were randomly divided into four groups. Control mdx aged between 4 and 26 weeks old were included that received no treatment. Treatment was initiated one week after birth by the addition of ectoine to drinking water (approximately 150 mg/kg) for two weeks. A group of mdx continued to receive ectoine in drinking water (approximately 1.08 g/kg), and another group from then on received daily intraperitoneal injections of ectoine (approximately 177 mg/kg). A treatment control group was administered saline via daily intraperitoneal injections. This study included both male and female mice, and a balanced number of each gender was used for the analyses.

At 5.5 weeks of age, cervical dislocation was performed. From the hind limbs, gastrocnemius (GAS), tibialis anterior (TA), soleus (SOL) and extensor digitorum longus (EDL) muscles were dissected and snap-frozen for Western blotting, or embedded in Tissue-Tek (Sakura Finetek Europe, Alphen aan den Rijn, The Netherlands) and submerged in nitrogen-cooled isopentane for immunofluorescence. All materials were stored at −80 °C until used.

### 4.2. Protein Sample Preparation

From the GAS muscle of 5.5-week-old mice and the EDL muscle of 26-week-old mice, total protein samples of individual mice were prepared. In the case of the EDL muscle, the size of the tissue of 5.5-week-old mice necessitated the pooling of the material from two individual mice per treatment group. Total protein was prepared by combining two EDL muscles per group in a glass–glass tissue grinder in an equal volume of extraction buffer (RIPA buffer (50 mM Tris–HCl, 150 mM NaCl, 2.5% NP40, 2.5% Na-deoxycholate, 0.1% sodium dodecyl sulphate pH 7.4)) supplemented with a complete protease inhibitor cocktail (Merck Life Science, Hoeilaart, Belgium). The samples were then subjected to centrifugation at 10,000× *g* for 15 min at 4 °C. The resulting supernatant was collected, and protein concentration was estimated based on the 260/280 nm absorbance using the Biodrop μLite device (Thermo Fisher Scientific, Waltham, MA, USA).

In order to obtain a sufficient protein yield, mitochondrial protein samples were prepared by pooling the SOL muscle from four mice per treatment group before preparation using a glass–glass tissue grinder. A published protocol of sequential centrifugation was employed [62], with certain modifications. In brief, SOL tissues were resolved in an STM buffer (250 mM sucrose, 50 mM Tris–HCl, 5 mM MgCl_2_ pH7.4), left on ice for 10 min and then centrifuged at 800× *g* for 20 min. The pellet was resuspended in an STM buffer and centrifuged for a further 10 min at 11,000× *g*. The pellet was resuspended in an SOL buffer (50 mM Tris–HCl, 1 mM EDTA, 0.5% Triton X-100 pH 6.8) and centrifuged for 10 min at 800× *g*. The supernatant was collected, representing the mitochondria-enriched protein fraction.

### 4.3. Western Blotting

Total protein samples were diluted to a protein concentration of 1500 μg/mL in lithium dodecyl sulfate buffer with a reducing agent added. Samples were boiled for 2 min, and 20 μL was loaded onto NuPAGE 10% Bis–Tris gels for electrophoresis in an MOPS buffer (except for mTOR visualization, which was done on a 3–8% Tris acetate gel more suited to larger proteins). The samples were then transferred to a nitrocellulose membrane via electroblotting (Thermo Fisher Scientific). The membrane was subjected to a sequential Western blotting protocol, whereby the protein of interest was imaged first via chemiluminescent detection, and the housekeeping protein glyceraldehyde-3-phosphate dehydrogenase (GAPDH) was imaged subsequently using chromogenic detection on the same blot. In order to achieve this, the membrane was incubated for 1 h with a milk blocking solution consisting of Tris-buffered saline with 0.1% Tween 20 (TBST) and 0.2% non-fat dry milk. The membrane was washed with TBST and incubated with primary antibodies in milk blocking solution overnight. The following primary antibodies and concentrations were used: 0.2 µg/mL rabbit monoclonal anti-ATG5 (D5F5U), 0.03 µg/mL rabbit monoclonal anti-ATG7 (D12B11), 0.04 µg/mL rabbit polyclonal anti-PRKN, 0.07 µg/mL rabbit monoclonal anti-SQSTM1 (D6M5X), 0.03 µg/mL rabbit polyclonal anti-p-SQSTM1, 0.1 µg/mL rabbit polyclonal anti-VDAC, 0.05 µg/mL rabbit monoclonal anti-PGC-1α (3G6), 0.1 µg/mL rabbit polyclonal anti-mTOR (Cell Signaling Technologies Europe, Leiden, The Netherlands), 0.9 µg/mL rabbit monoclonal anti-LC3 II (ab192890) (Abcam, Trumpington, UK) and 2 µg/mL rabbit polyclonal anti-HSP70 (R&D Systems, Minneapolis, MN, USA). The membranes were then washed with TBST and incubated with 0.5 µg/mL horseradish peroxidase-labeled goat anti-rabbit secondary antibody (Sigma Aldrich, St Louis, MO, USA) for 1 h. Protein bands were visualized using a chemiluminescent ECL Pierce substrate (Thermo Fisher Scientific) using the Chemidoc Imaging System (Bio-Rad Laboratories, Temse, Belgium). Subsequently, the same blot was incubated with 0.2 µg/mL rabbit polyclonal anti-GAPDH (ab9485) (Abcam) for 1 h, and protein bands were detected with alkaline phosphatase-linked anti-rabbit secondary antibody using the Western Breeze kit (Thermo Fisher Scientific). The density of protein bands was quantified with Gene Tools software version 4.0. (Syngene, Cambridge, UK) by calculating the ratio relative to GAPDH levels to correct for protein loading differences between samples.

The standard housekeeping protein for the normalization of mitochondrial protein levels, VDAC, has been proposed to serve as a mitochondrial docking site to recruit PRKN from the cytosol to defective mitochondria [63]. We therefore elected not to utilize VDAC protein levels for normalization, but rather to employ normalization to total protein. Mitochondrial protein samples were resolved in a Laemmli buffer containing 2-mercaptoethanol, boiled for 2 min and loaded onto 4–20% Mini-Protean TGX stain-free gels for electrophoresis. Proteins were transferred to ethanol-treated low-fluorescent polyvinylidene membranes (Bio-Rad Laboratories) and incubated overnight with primary antibodies at the same concentrations as specified for total protein samples. Protein bands were visualized with horseradish peroxidase-conjugated secondary antibodies (Sigma Aldrich) and chemiluminescent ECL Pierce substrate (Thermo Fisher Scientific) using the Chemidoc Imaging System (Bio-Rad Laboratories). Densities were quantified with Image-Lab 6.0 software and normalized to total protein using stain-free technology (Bio-Rad Laboratories).

### 4.4. Statistical Analyses

Statistical analyses were performed using GraphPad Prism software version 10.2.3. (GraphPad Software, Boston, MA, USA). For the comparison of two independent measurements with normal distribution, the unpaired *t* test was used. For three and four sets of unpaired measurements with Gaussian distribution and unequal variances, the Brown–Forsythe and Welch ANOVA tests for multiple comparisons were used.

### 4.5. Immunofluorescence Staining

Eight µm frozen sections were cut from TA and SOL muscles. The sections were permeabilized for 2 min in cooled acetone and were incubated in phosphate-buffered saline (PBS) with 2% bovine serum albumin, 10% heat-inactivated human serum and 5% donkey serum (PBS/BSA/HD) for 30 min. The primary antibodies were diluted in PBS/BSA/HD and incubated for 3 h with the following concentrations of antibodies: 1.1 µg/mL rabbit monoclonal anti-ATG5 (D5F5U), 0.2 µg/mL rabbit monoclonal anti-ATG7 (D12B11), 0.9 µg/mL rabbit monoclonal anti-ATG16L1 (D6D5), 0.2 µg/mL rabbit polyclonal anti-PRKN, 1 µg/mL rabbit monoclonal anti-LC3 II (D11), 0.5 µg/mL rabbit monoclonal anti-SQSTM1 (D6M5X) (Cell Signaling Technologies), 10 µg/mL rabbit polyclonal anti-HSP70 (R&D Systems), 5 µg/mL rat anti-F4/80 (Abcam) and 2.5 µg/mL goat polyclonal anti-NCAM (R&D Systems). Sections were rinsed three times with PBS and incubated for 30 min with secondary antibodies: 0.25 µg/mL donkey anti-rabbit AlexaFluor 488 conjugated (Thermo Fisher Scientific) and 0.25 µg/mL donkey anti-goat CY3 conjugated (Jackson Laboratories, West Grove, PA, USA) diluted in PBS. The sections were then rinsed three times with PBS and mounted with Fluoromount G (Thermo Fisher Scientific). Stained sections were visualized under a fluorescent microscope (Carl Zeiss, Jena, Germany) and imaged using Cell F software version 3.4. (Olympus Life Science, Antwerp, Belgium).

## 5. Conclusions

Our study revealed the altered expression of autophagy-related proteins in the muscles of ectoine-treated mdx, which reinforces the notion that the compound could be tested further as a viable therapeutic intervention for DMD. Caution is, however, due when interpreting the dynamic process of autophagy through the interpretation of results obtained from static snapshots. Whether the administration of osmolytes as supplements may be an effective method for enhancing autophagic efficiency and the subsequent improved clearance of defective cellular components in human DMD would need to be investigated. Given that autophagic deficits are complicators of tissue recovery in various neuromuscular disorders [64], including also muscular dystrophies due to *COL6* [65] and *DYSF* [66] deficiencies, the expansion of the compound’s potential applicability could also reach beyond DMD treatment.

## Figures and Tables

**Table 1:** Comparison of microtubule-associated protein 1A/1B-light chain 3 and sequestosome 1 protein levels between healthy mice (BL 10) and mdx mice.

Marker	Muscle	Age	Regulation	Reference
LC3 I	quadriceps	11 weeks	mdx > BL 10	[42]
	gastrocnemius	11 months	D2-mdx > BL 10	[43]
	diaphragm	11 months	D2-mdx > BL 10	[43]
	heart	7 weeks	mdx ≅ BL 10	[44]
	heart	5.5 months	mdx > BL 10	[45]
	heart	14 months	mdx ≅ BL 10	[44]
	heart	17 months	mdx ≅ BL 10	[44]
LC3 II	quadriceps	11 weeks	mdx > BL 10	[42]
	tibialis anterior	5.5 months	mdx > BL 10	[46]
	extensor digitorum longus	5.5 weeks	mdx* ≅ BL 10	this study
	gastrocnemius	5.5 weeks	mdx* ≅BL 10	this study
	gastrocnemius	11 months	D2-mdx ≅ BL 10	[43]
	diaphragm	11 months	D2-mdx ≅ BL 10	[43]
	heart	7 weeks	mdx ≅ BL 10	[44]
	heart	5.5 months	mdx > BL 10	[45]
	heart	12 months	mdx > BL 10	[47]
	heart	14 months	mdx < BL 10	[44]
	heart	17 months	mdx < BL 10	[44]
LC3 II/I	quadriceps	11 weeks	mdx ≅ BL 10	[42]
	quadriceps	5.5 months	mdx > BL 10	[14]
	tibialis anterior	4 months	mdx > BL 10	[48]
	extensor digitorum longus	6.5 months	mdx > BL 10	this study
	gastrocnemius	11 months	D2-mdx ≅ BL 10	[43]
	soleus	3.5 months	mdx < BL 10	[49]
	diaphragm	6 weeks	mdx > BL 10	[50]
	diaphragm	3 months	mdx > BL 10	[50]
	diaphragm	4 months	mdx > BL 10	[48]
	diaphragm	11 months	D2-mdx ≅ BL 10	[43]
	heart	14 months	mdx ≅ BL 10	[44]
SQSTM1	quadriceps	11 weeks	mdx ≅ BL 10	[42]
	quadriceps	5.5 months	mdx > BL 10	[14]
	tibialis anterior	4 months	mdx > BL 10	[48]
	tibialis anterior	5.5 months	mdx > BL 10	[46]
	extensor digitorum longus	5.5 weeks	mdx* ≅ BL 10	this study
	gastrocnemius	5.5 weeks	mdx* ≅ BL 10	this study
	gastrocnemius	11 months	D2-mdx < BL 10	[43]
	soleus	3.5 months	mdx > BL 10	[49]
	diaphragm	4 months	mdx > BL 10	[48]
	diaphragm	11 months	D2-mdx > BL 10	[43]
	heart	7 weeks	mdx ≅ BL 10	[44]
	heart	5.5 months	mdx > BL 10	[45]
	heart	12 months	mdx > BL 10	[47]
	heart	14 months	mdx ≅ BL 10	[44]
	heart	17 months	mdx ≅ BL 10	[44]

Abbreviations: microtubule-associated protein 1A/1B-light chain 3 unconjugated (LC3 I) and conjugated to phosphatidylethanolamine (LC3 II); sequestosome 1 (SQSTM1); mdx crossed into the DBA/2J genetic background (D2-mdx); mdx treated with intraperitoneal saline (mdx*).

## Data Availability

The raw data presented in this study are available from the corresponding author on reasonable request.

## Supplementary Materials

The following supporting information can be downloaded at https://www.mdpi.com/article/10.3390/ijms26020439/s1.
