# Supplementary material for: Changes to the Autophagy-Related Muscle Proteome Following Short-Term Treatment with Ectoine in the Duchenne Muscular Dystrophy Mouse Model mdx
Source: Int J Mol Sci. 2025 Jan 7;26(2):439. doi: 10.3390/ijms26020439 (PMC11765399; doi:10.3390/ijms26020439)

Figure S1: Full blots for protein band panels of Figures 1,3,4 and 6

Figure 1 Full blots

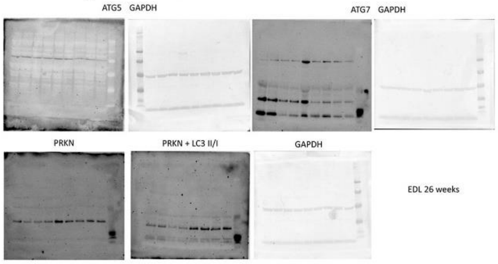

Figure 3 Full blots

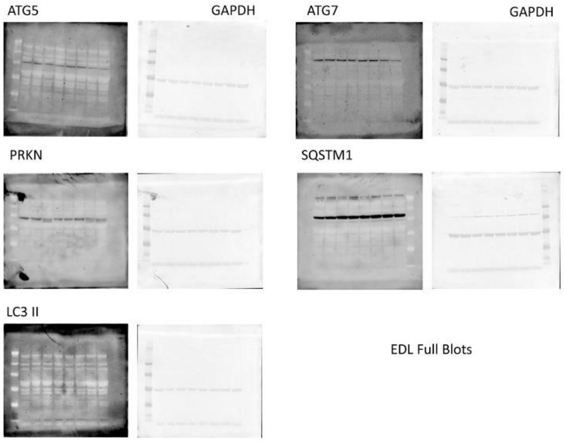

Figure 4 Full blots

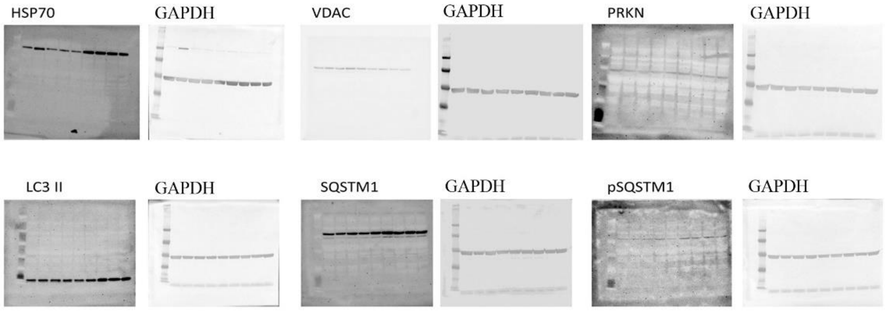

GAS Full Blots

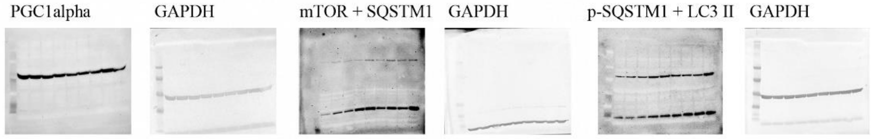

Figure 6 Full blots

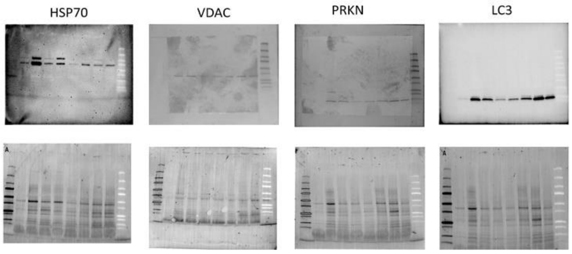

Figure S2: Immunofluorescence staining of tibialis anterior muscle of 12-weeks old mice

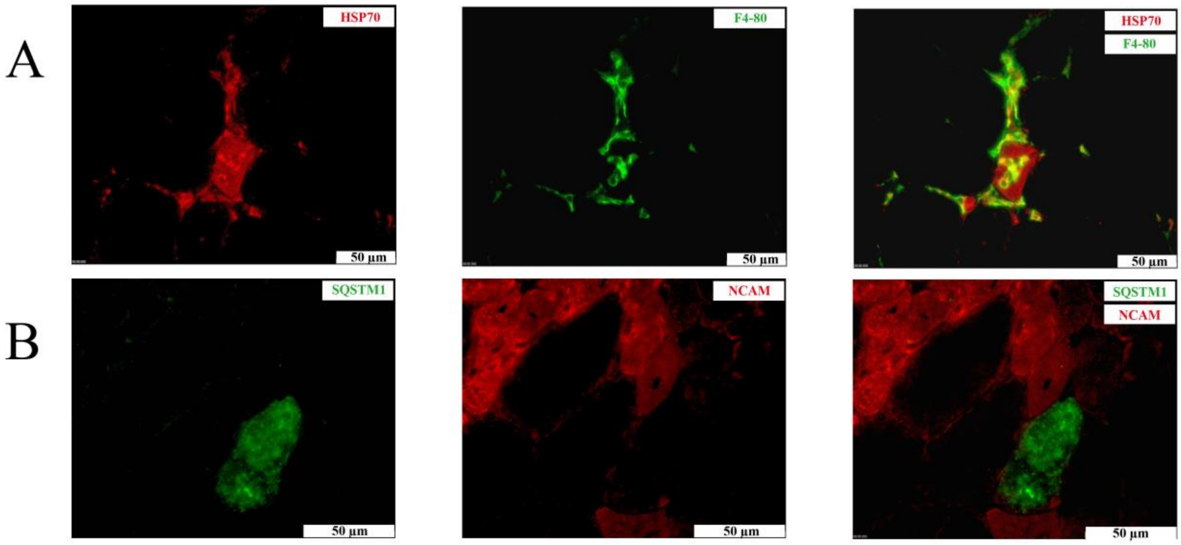

(**A**) Strong sarcoplasmic heat shock protein 70 (HSP70) staining (CY3, red) is observed in a necrotic muscle fiber. Macrophages are identified as F4-80 positive (AlexaFluor 488, green). Double staining shows HSP70 positivity in the muscle fiber's sarcoplasm and in part of the macrophages. (**B**) The sarcoplasm of a muscle fiber stains strongly for SQSTM1 (AlexaFluor 488, green). Several NCAM positive (CY3, red) muscle fibers can be observed in the vicinity, however, the SQSTM1 positive muscle fiber is NCAM negative. Scale bars=50µm.

Figure S3: Quantification of GAPDH levels over total protein in BL 10 and mdx

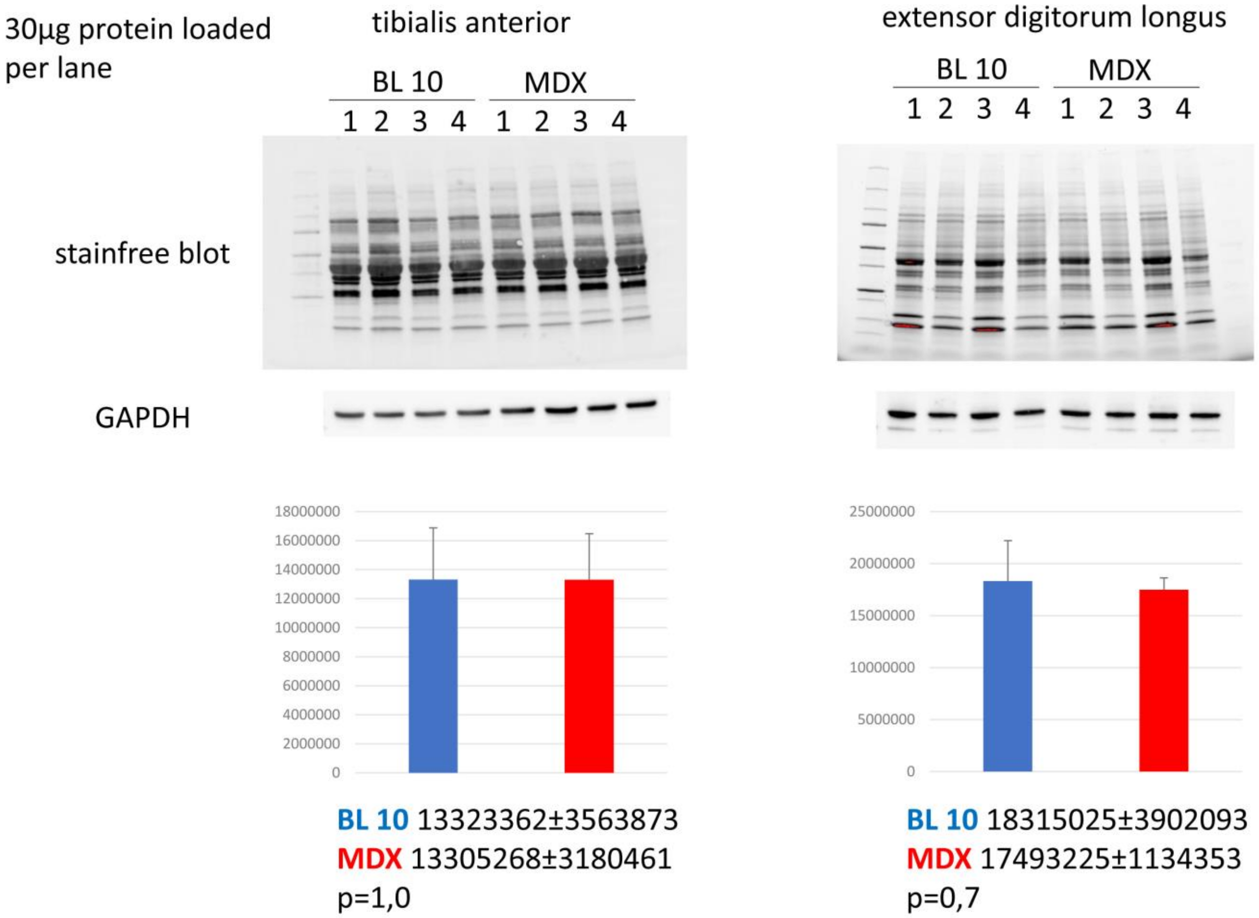

Supplement: Supplementary file 1 [file ijms-26-00439-s001.zip › ijms-3345285-supplementary.pdf]
